# Supplementary material for: Effect of elevation, season and accelerated snowmelt on biogeochemical processes during isolated conifer needle litter decomposition
Source: PeerJ. 2021 Aug 10;9:e11926. doi: 10.7717/peerj.11926 (PMC8362670; doi:10.7717/peerj.11926)
Supplement: Supplemental Information 1 — Snow-free dates determined by the first spring date when soil microclimate temperatures were consistently > 1 degree Celsius. These dates are estimates, as more specific dates would be determined using soil temperature data with time-lapse photos to determine when the complete plot area was snow-free. [file peerj-09-11926-s001.docx]

| **Lower** | **Middle-ES** | **Middle** | **Upper** |
| --- | --- | --- | --- |
| 5/15/17 | 5/14/17 | 6/5/17 | 6/27/17 |
| 4/29/18 | 4/29/18 | 5/13/18 | 6/1/18 |
| 5/15/19 | 6/7/19 | 6/15/19 | - |
