# Supplementary figures and images for: Effect of elevation, season and accelerated snowmelt on biogeochemical processes during isolated conifer needle litter decomposition
Source: PeerJ. 2021 Aug 10;9:e11926. doi: 10.7717/peerj.11926 (PMC8362670; doi:10.7717/peerj.11926)

# Butte 380

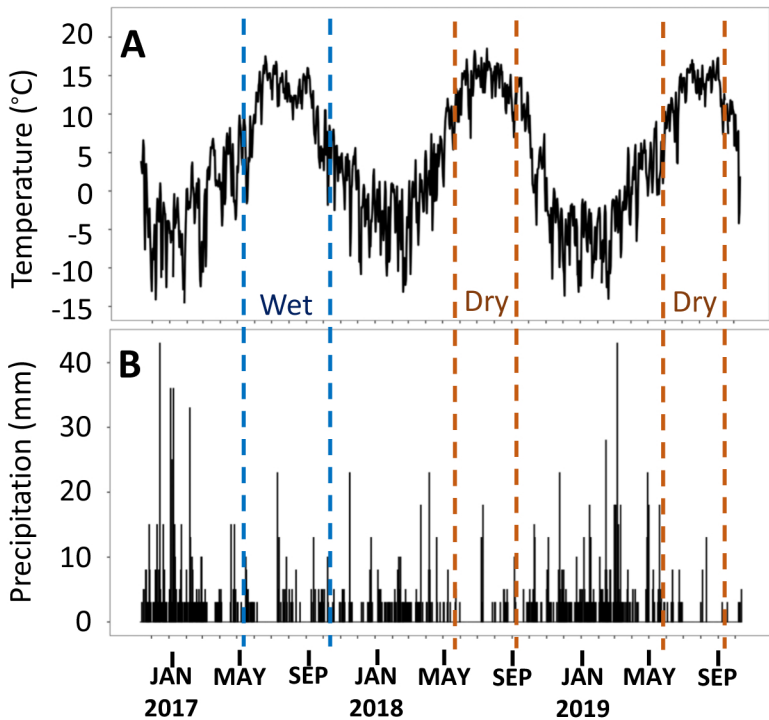

Supplement: Supplemental Information 2 — Local air temperature and total precipitation are shown from the Butte 380 USDA SNOTEL station. [file peerj-09-11926-s002.pdf]

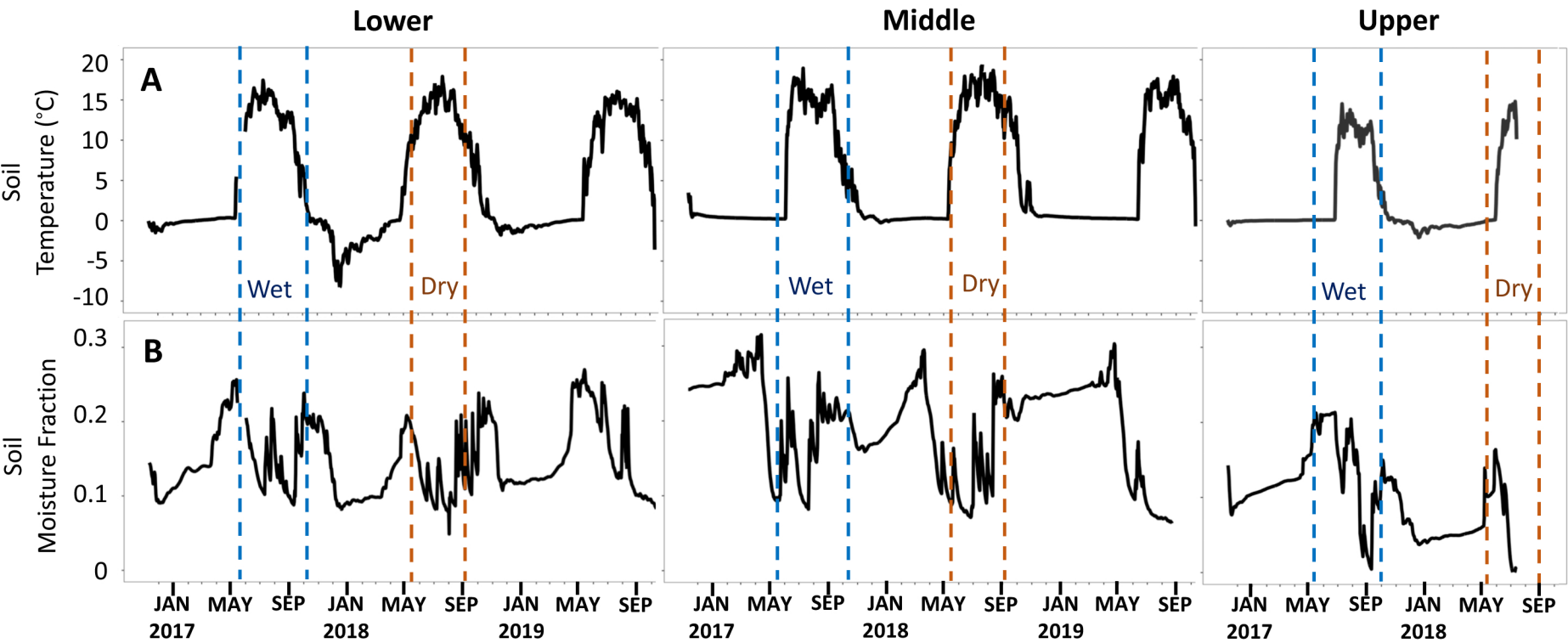

Supplement: Supplemental Information 3 — (A) soil temperature and (B) soil moisture are shown for each study elevation at the Lower (2,800 m), Middle (3,100 m), and Upper (3,500 m) plots. Note microclimate data collection ceased in Fall 2018 at the Upper plot. [file peerj-09-11926-s003.pdf]

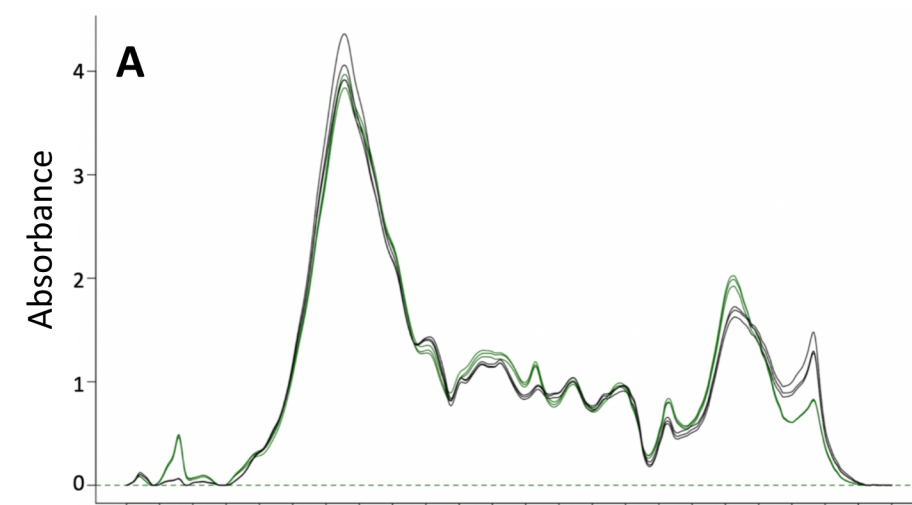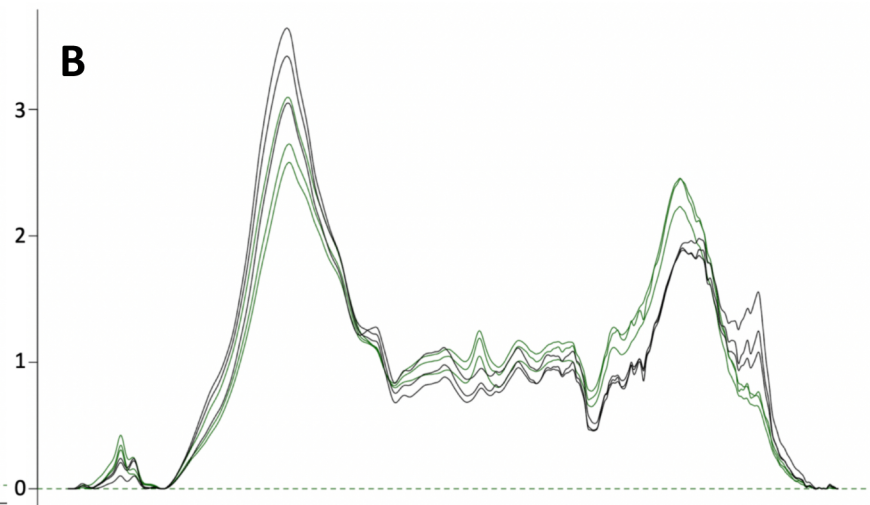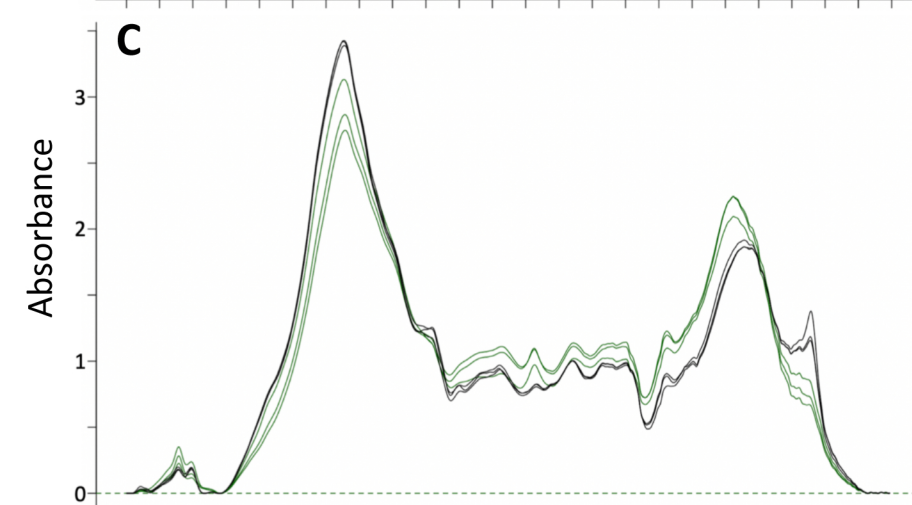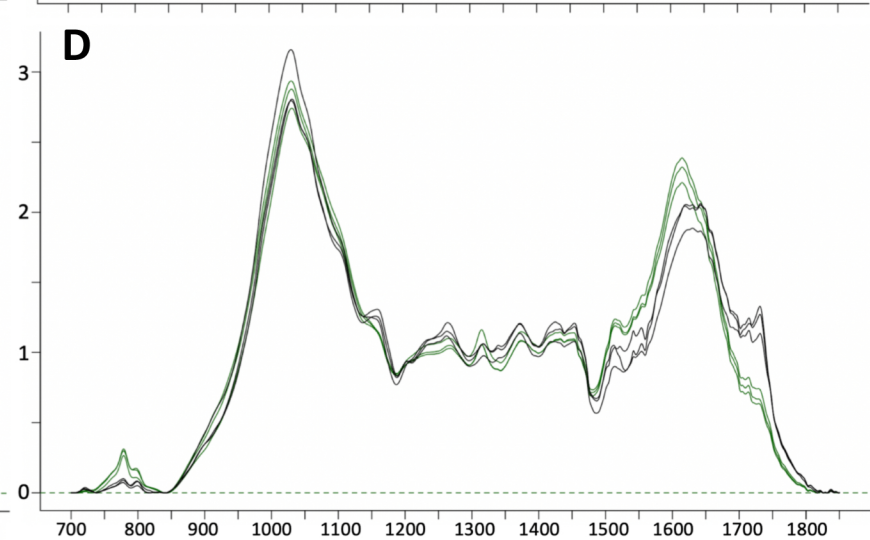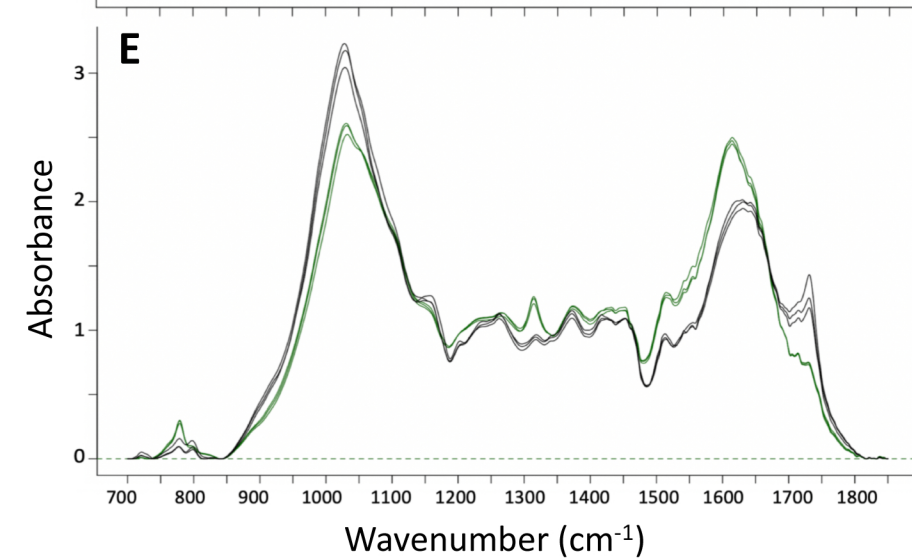

— Spruce  
— Lodgepole

Supplement: Supplemental Information 6 — (A) The harvested needles FTIR spectra are shown against (B) Lower (2,800 m), (C) Middle (3,100 m), (D) Middle-ES (3,100 m), and (E) Upper (3,500 m) plots. [file peerj-09-11926-s006.pdf]

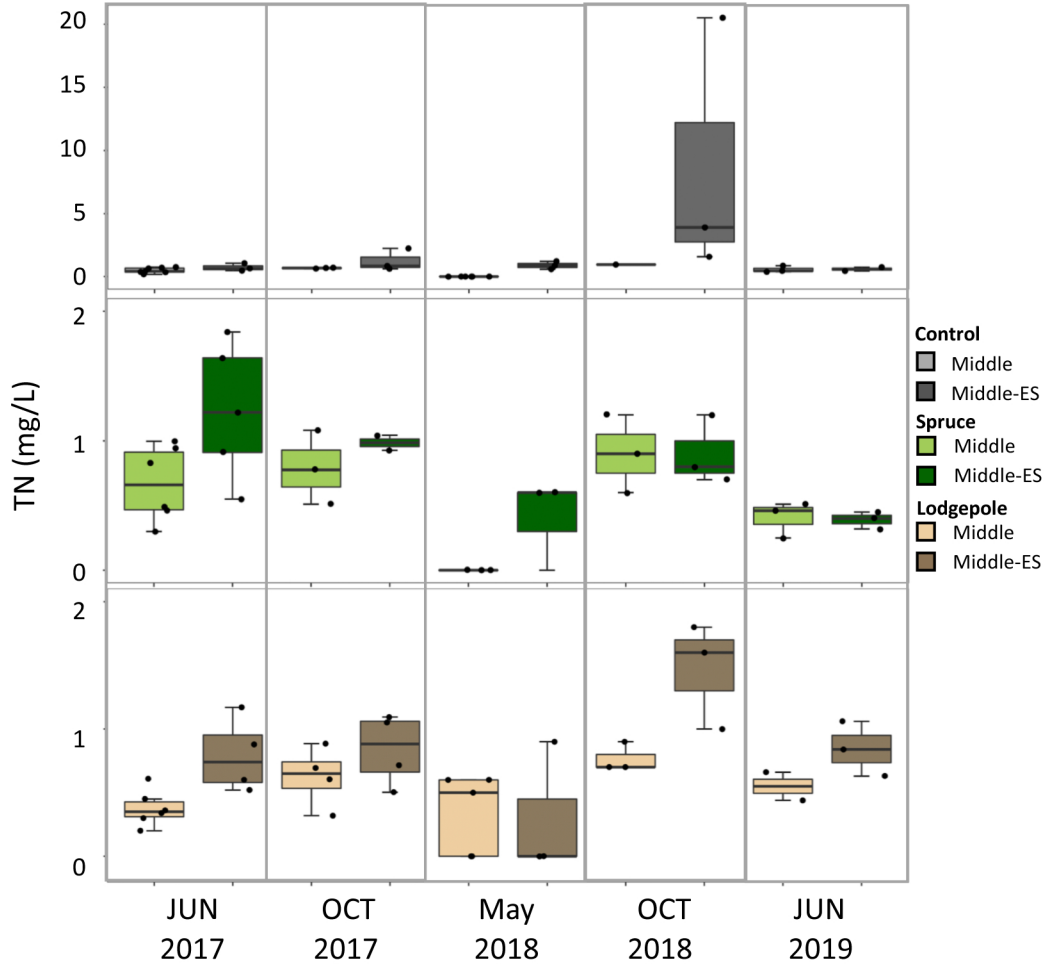

Supplement: Supplemental Information 8 — 2017-2019 concentrations at the Lower Subalpine control (Middle) and manipulated (Middle-ES) plots for the control, spruce, and lodgepole samples. Error bars indicate plus or minus one standard deviation. Samples were collected during two consecutive days in 2017-2018 to increase the sample size (n=3-6). Individual points represent each sample measurement. [file peerj-09-11926-s008.pdf]

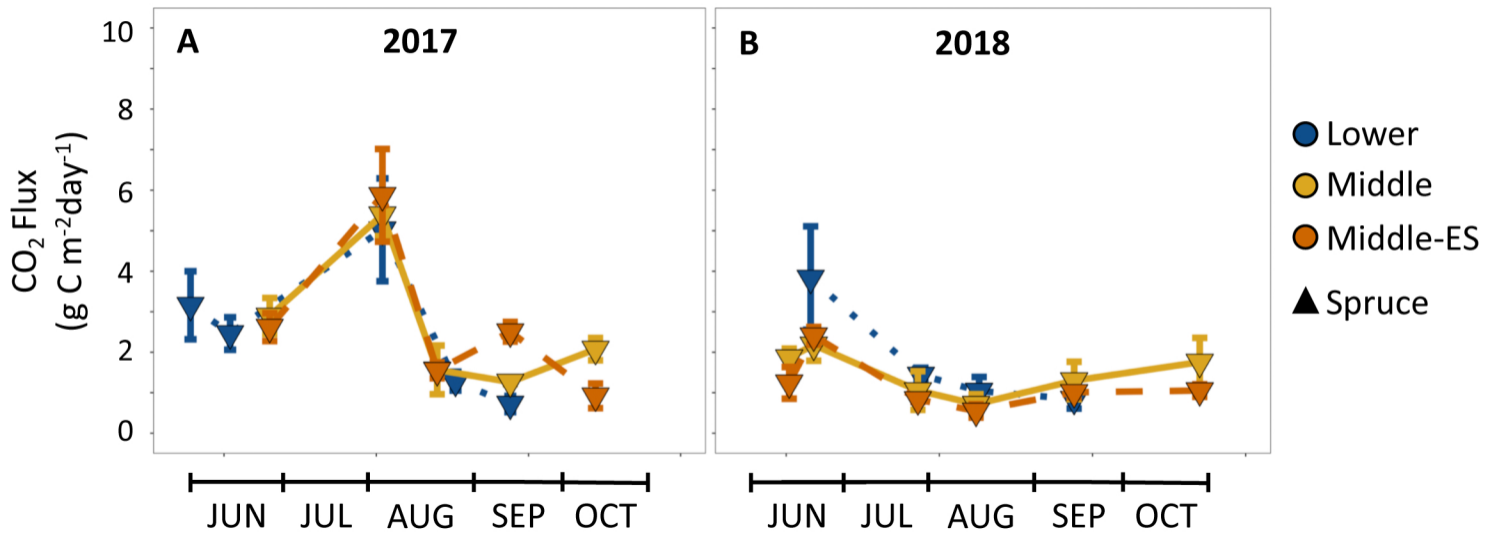

Supplement: Supplemental Information 9 — CO2 flux measurements over the snow-free months of (A) 2017 and (B) 2018 under spruce needles across elevation and snowmelt manipulation. [file peerj-09-11926-s009.pdf]

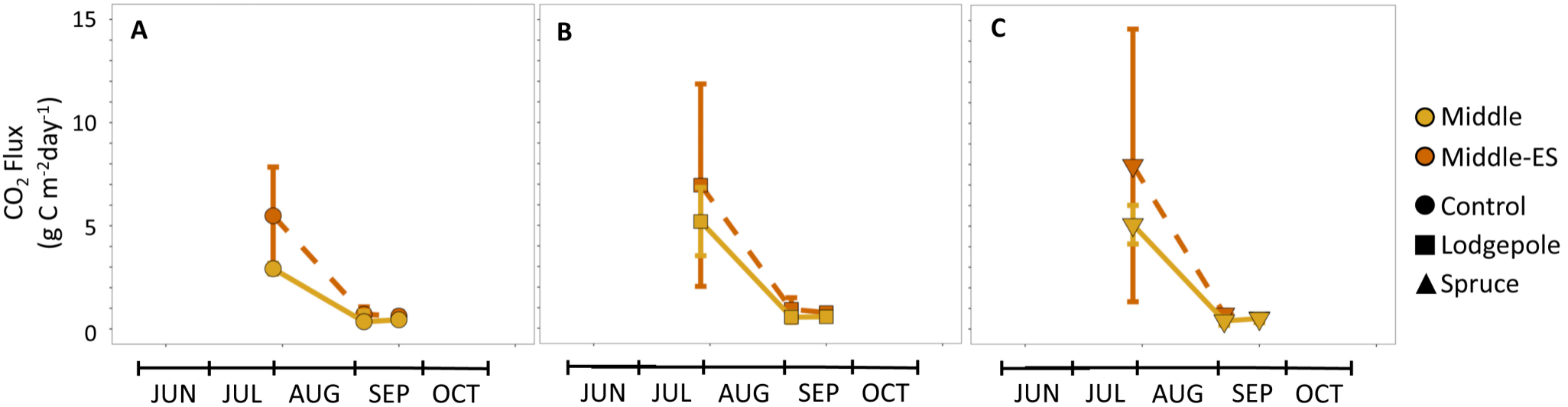

Supplement: Supplemental Information 10 — CO2 flux measurements over the snow-free months of 2019 under (A) control, (B) lodgepole, and (C) spruce samples across snowmelt manipulations. Lower plot measurements were not conducted in 2019. [file peerj-09-11926-s010.pdf]

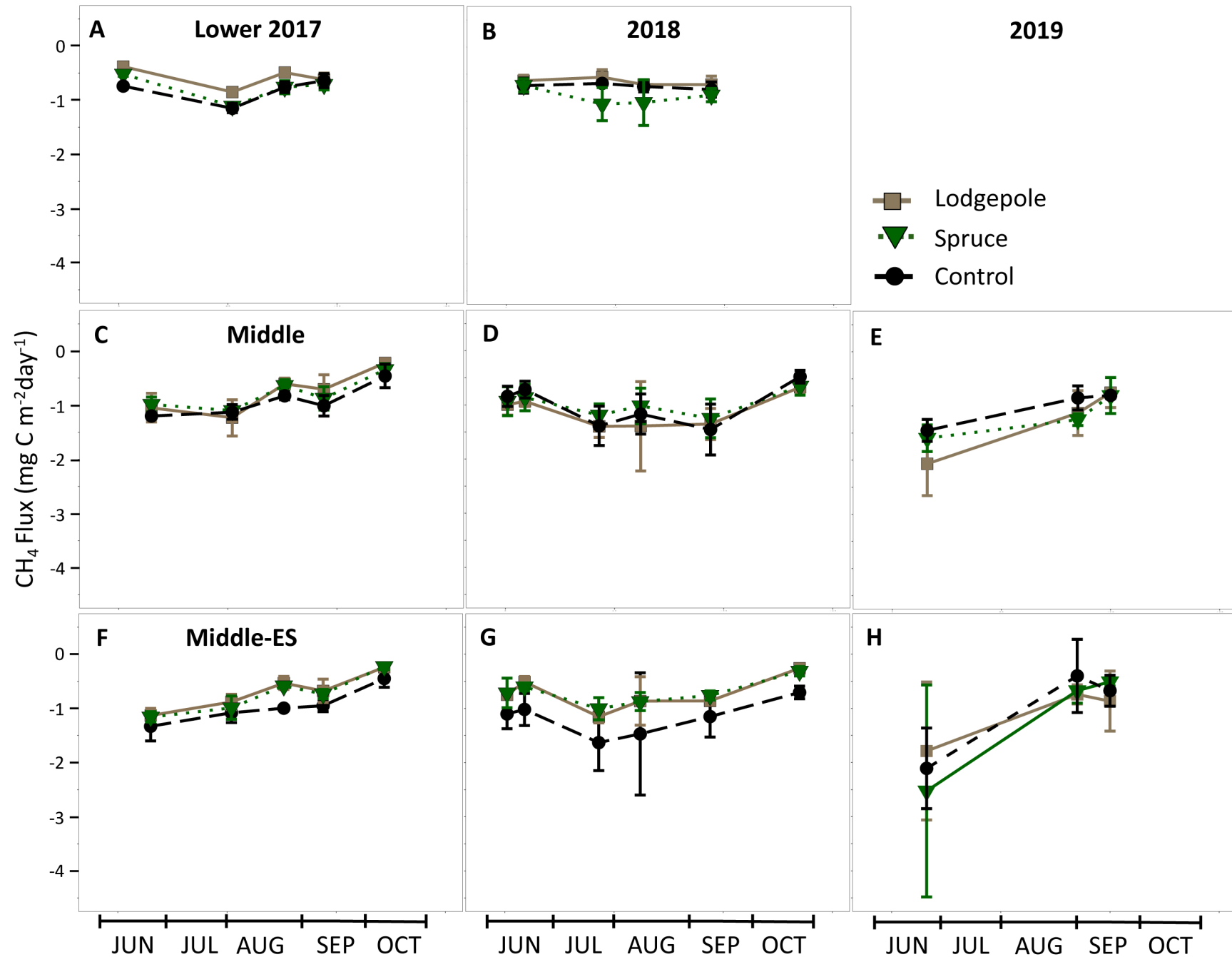

Supplement: Supplemental Information 12 — CH4 flux measurements over the snow-free months of 2017-2019 at the (A, B) Lower, (C, D, E) Middle, and (F, G, H) Middle-ES plots. Error bars indicate plus or minus one standard deviation. [file peerj-09-11926-s012.pdf]

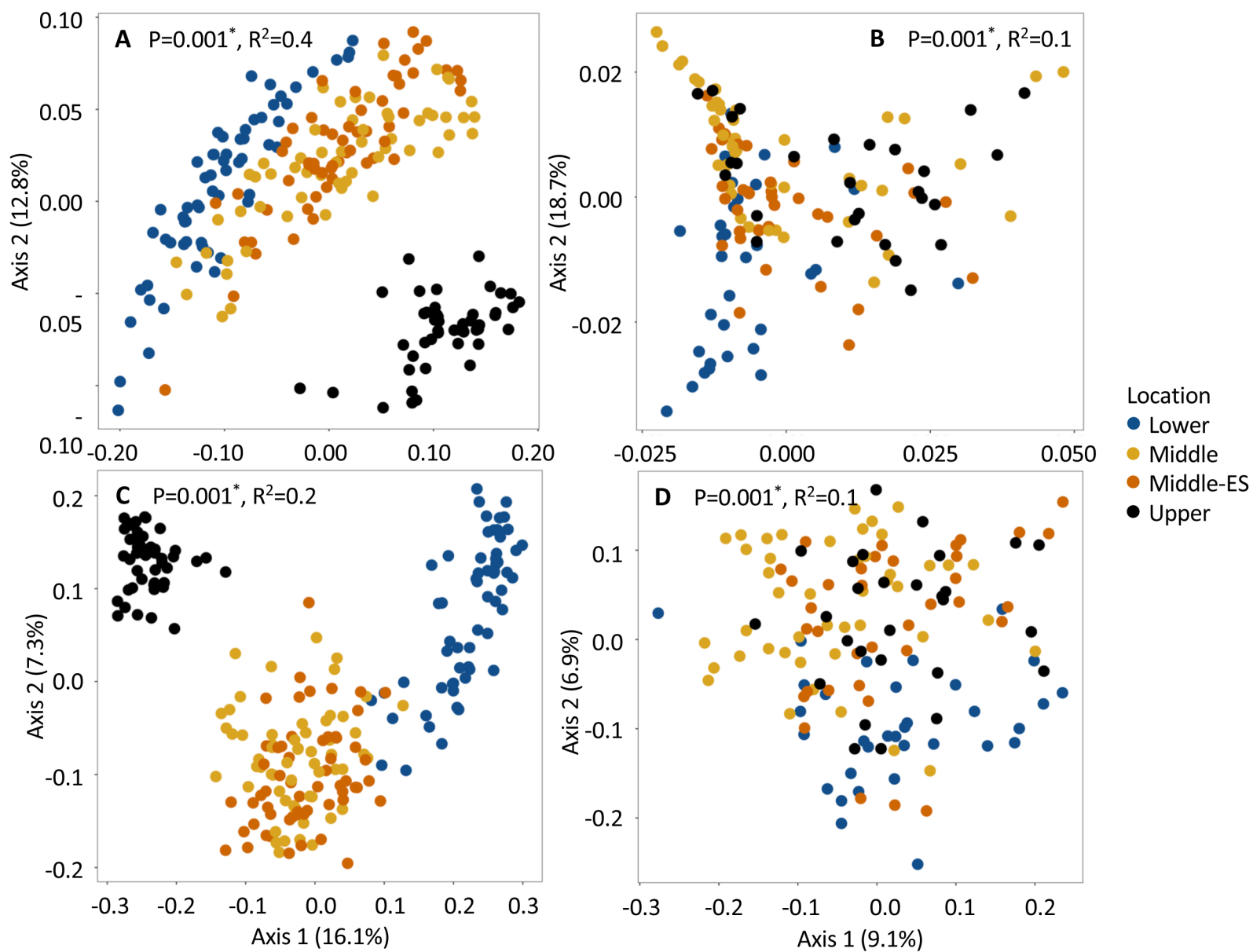

Supplement: Supplemental Information 13 — All the samples collected over time are shown for (A, C) 16S and (B, D) 18S communities as (A, B) weighted and (C, D) unweighted. P and R values represent ADONIS significance aggregated by location. [file peerj-09-11926-s013.pdf]

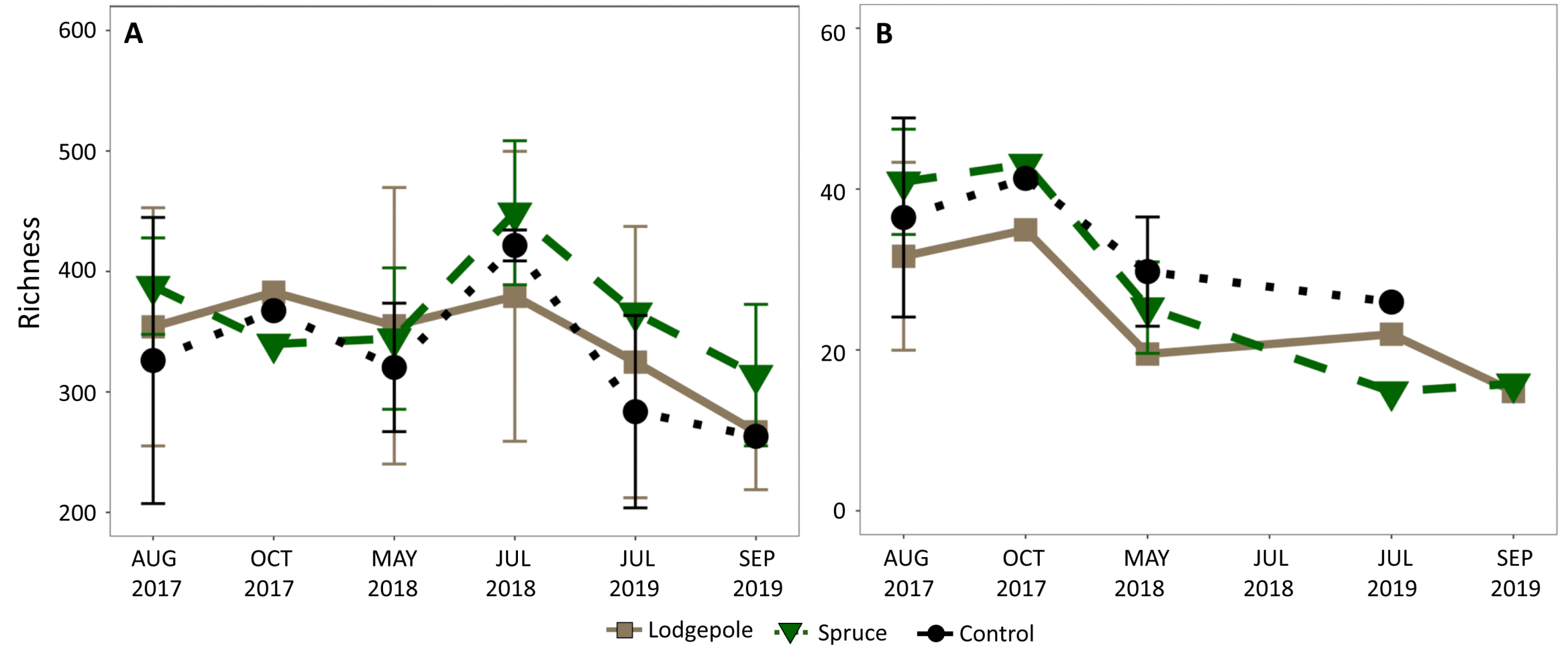

Supplement: Supplemental Information 15 — Species richness for the Upper (A) 16S and (B) 18S plots. Error bars indicate plus or minus one standard deviation (n=3, 4). Note a lack of error bars for a sample point is due to n<3 caused by sample reduction during rarefaction. [file peerj-09-11926-s015.pdf]

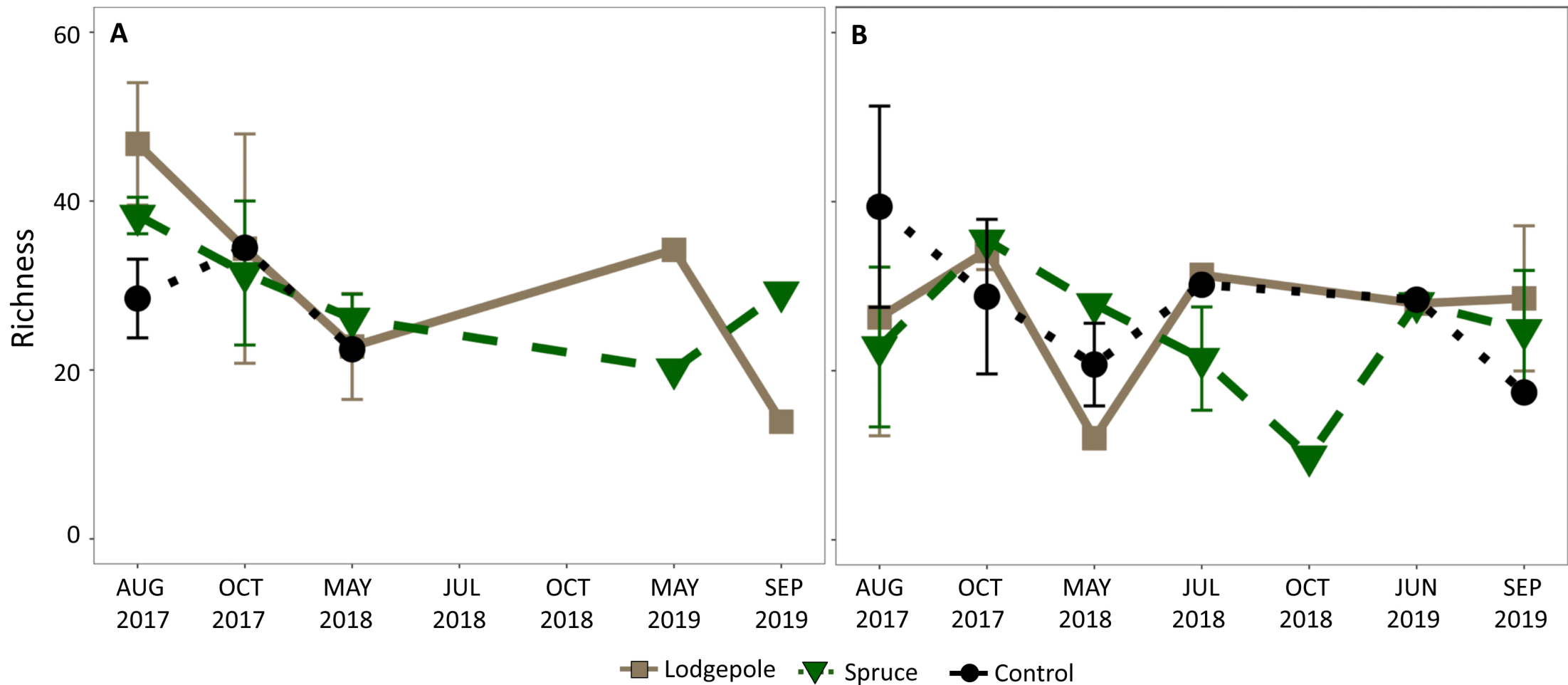

Supplement: Supplemental Information 17 — Species richness at the (A) Lower 18S and (B) Middle 18S plots. Error bars indicate plus or minus one standard deviation (n=3, 4). Note a lack of error bars for a sample point is due to n<3 caused by sample reduction during rarefaction. [file peerj-09-11926-s017.pdf]

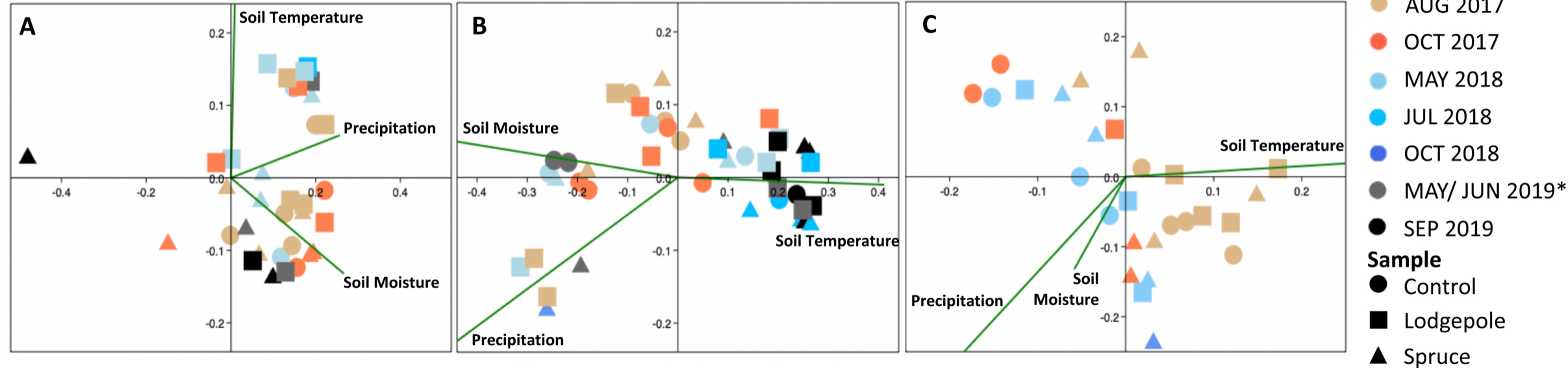

Supplement: Supplemental Information 19 — Canonical correspondence analysis of environmental variables driving beta diversity community clustering as a function of soil temperature and moisture and precipitation over time at the (A) Lower, (B) Middle, and (C) Middle-ES plots *The early 2019 sampling dates were dependent on snowpack, with the Lower samples collected in May 2019, and Middle in June 2019. [file peerj-09-11926-s019.pdf]
