# Supplementary material for: Effect of elevation, season and accelerated snowmelt on biogeochemical processes during isolated conifer needle litter decomposition
Source: PeerJ. 2021 Aug 10;9:e11926. doi: 10.7717/peerj.11926 (PMC8362670; doi:10.7717/peerj.11926)
Supplement: Supplemental Information 4 — Percent mass losses for the spruce and lodgepole needle litter bags. Note the lodgepole at the Lower site has no standard deviation due to n=2. Litter bags at the Upper site were displaced by cattle. [file peerj-09-11926-s004.docx]

|  | **Lower (%)** | **Middle-ES (%)** | **Middle (%)** |
| --- | --- | --- | --- |
| Spruce | 17 (±1) | 17 (±3) | 13 (±4) |
| Lodgepole | 21 - | 22 (±2) | 21 (±6) |
