# Supplementary material for: Effect of elevation, season and accelerated snowmelt on biogeochemical processes during isolated conifer needle litter decomposition
Source: PeerJ. 2021 Aug 10;9:e11926. doi: 10.7717/peerj.11926 (PMC8362670; doi:10.7717/peerj.11926)
Supplement: Supplemental Information 5 [file peerj-09-11926-s005.docx]

| **2016 Initial Needle Litter** | | | |  | |  | |  | |  | |  | |  | | | |  | |  | |  | |  | |  | | | |  | |  | | |  | |  |  |
| --- | --- | --- | --- | --- | --- | --- | --- | --- | --- | --- | --- | --- | --- | --- | --- | --- | --- | --- | --- | --- | --- | --- | --- | --- | --- | --- | --- | --- | --- | --- | --- | --- | --- | --- | --- | --- | --- | --- |
|  | Spruce | Lodgepole |  | |  | |  | |  | |  | |  | | | |  | |  | |  | |  | |  | | | |  | |  | | |  | |  |  |  |
| C (%) | 45.9 (±0.2) | 53.7 (±0.1) |  | |  | |  | |  | |  | |  | | | |  | |  | |  | |  | |  | | | |  | |  | | |  | |  |  |  |
| N ( %) | 0.7 (±2E-2) | 0.9 (±2E-2) |  | |  | |  | |  | |  | |  | | | |  | |  | |  | |  | |  | | | |  | |  | | |  | |  |  |  |
| C:N | 62.7 (±1.7) | 61.6 (±1.1) |  | |  | |  | |  | |  | |  | | | |  | |  | |  | |  | |  | | | |  | |  | | |  | |  |  |  |
| **2019 Lower** | | | | **2019 Middle** | | | | | | | | | | | | **2019 Middle-ES** | | | | | | | | | | | | **2019 Upper** | | | | | | | | | | |
|  | Spruce | Lodgepole | Spruce | | | | | | Lodgepole | | | | | | Spruce | | | | | | Lodgepole | | | | | | Spruce | | | | | | Lodgepole | | | | |  |
| C (%) | 41.6 (±7.8) | 50.7 (±5.5) | 42.4 (±3.8) | | | | | | 47.4 (±2.2) | | | | | | 44.5 (±2.6) | | | | | | 50.2 (±1.0) | | | | | | 50.0 (±4.1) | | | | | | 49.3 (±1.3) | | | | |  |
| N (%) | 0.9 (±0.1) | 1.0 (±0.1) | 1.1 (±0.1) | | | | | | 0.9 (±0.1) | | | | | | 1.1 (±9E-3) | | | | | | 1.0 (±2E-2) | | | | | | 1.1 (±0.1) | | | | | | 1.1 (±0.1) | | | | |  |
| C:N | 43.7 (±1.5) | 48.4 (±2.4) | 39.5 (±1.7) | | | | | | 50.3 (±6.4) | | | | | | 40.4 (±2.7) | | | | | | 51.9 (±1.0) | | | | | | 43.8 (±0.6) | | | | | | 46.3 (±3.5) | | | | |  |
