# Supplementary material for: Effect of elevation, season and accelerated snowmelt on biogeochemical processes during isolated conifer needle litter decomposition
Source: PeerJ. 2021 Aug 10;9:e11926. doi: 10.7717/peerj.11926 (PMC8362670; doi:10.7717/peerj.11926)
Supplement: Supplemental Information 7 [file peerj-09-11926-s007.docx]

|  | **Polysaccharides**  **(900-1200 cm^-1^)^1^** | | **Ether Linkage**  **(1150 cm^-1^)^2^** | | **Carbonyl**  **(1738 cm^-1^) ^2^** | | **Amide I**  **(1600-1700 cm^-1^)^2^** | | **Aromatics**  **(1508-1510 cm^-1^)^1^** | | **Polysaccharides to Amide I Ratio** | |
| --- | --- | --- | --- | --- | --- | --- | --- | --- | --- | --- | --- | --- |
| **Spruce 2016** | | | | | | | | | | | | |
| **Harvested Spruce** | 529 | (±3) | 50 | (±2) | 38 | (±1) | 193 | (±4) | 33 | (±1) | 2.7 | (±0.1) |
| **Spruce 2019** | | | | | | | | | | | | |
| Lower | 425 | (±41) | 51 | (±1) | 41 | (±3) | 302 | (±17) | 51 | (±6) | 1.4 | (±0.2) |
| Middle | 440 | (±33) | 54 | (±1) | 43 | (±5) | 290 | (±9) | 47 | (±3) | 1.5 | (±0.2) |
| Middle-ES | 430 | (±20) | 52 | (±1) | 40 | (±3) | 295 | (±11) | 53 | (±1) | 1.5 | (±0.1) |
| Upper | 389 | (±5) | 52 | (±1) | 43 | (±1) | 315 | (±7) | 54 | (±3) | 1.3 | (±0.0) |
| **Lodgepole 2016** | | | | | | | | | | | | |
| **Harvested Lodgepole** | 535 | (±18) | 64 | (±2) | 72 | (±6) | 180 | (±6) | 19 | (±1) | 3.0 | (±0.2) |
| **Lodgepole 2019** | | | | | | | | | | | | |
| Lower | 487 | (±45) | 59 | (±4) | 66 | (±13) | 258 | (±8) | 34 | (±2) | 1.9 | (±0.2) |
| Middle | 470 | (±50) | 57 | (±2) | 63 | (±2) | 271 | (±25) | 36 | (±5) | 1.8 | (±0.3) |
| Middle-ES | 453 | (±49) | 60 | (±3) | 69 | (±1) | 265 | (±17) | 34 | (±7) | 1.7 | (±0.3) |
| Upper | 450 | (±12) | 59 | (±3) | 67 | (±6) | 269 | (±4) | 35 | (±2) | 1.7 | (±0.1) |

^1^Özgenç, Durmaz & Kuştaş, 2017

^2^Naumann, 2000
