# Supplementary material for: Effect of elevation, season and accelerated snowmelt on biogeochemical processes during isolated conifer needle litter decomposition
Source: PeerJ. 2021 Aug 10;9:e11926. doi: 10.7717/peerj.11926 (PMC8362670; doi:10.7717/peerj.11926)
Supplement: Supplemental Information 11 [file peerj-09-11926-s011.docx]

| **Lower** | | | | | | | | |
| --- | --- | --- | --- | --- | --- | --- | --- | --- |
|  | **CO_2_ and Temperature** | | **CO_2_ and Moisture** | | **CH_4_ and Temperature** | | **CH_4_ and Moisture** | |
|  | **P Value** | **R Value** | **P Value** | **R Value** | **P Value** | **R Value** | **P Value** | **R Value** |
| Control | **<0.01** | **0.59** | **<0.01** | **0.82** | 0.32 | -0.19 | 0.44 | 0.14 |
| Spruce | **<0.01** | **0.62** | **<0.01** | **0.62** | 0.35 | -0.18 | **<0.01** | **0.59** |
| Lodgepole | **<0.01** | **0.64** | **<0.01** | **0.49** | 0.36 | 0.17 | **<0.01** | **0.61** |
| **Middle** | | | | | | | | |
|  | **P Value** | **R Value** | **P Value** | **R Value** | **P Value** | **R Value** | **P Value** | **R Value** |
| Control | 0.21 | 0.19 | 0.47 | 0.11 | **<0.01** | **-0.48** | 0.51 | -0.10 |
| Spruce | 0.78 | -0.04 | 0.25 | 0.18 | **<0.01** | **-0.57** | **<0.01** | **-0.46** |
| Lodgepole | 0.50 | 0.10 | 0.37 | 0.14 | **<0.01** | **-0.54** | **<0.01** | **-0.41** |
| **Middle-ES** | | | | | | | | |
|  | **P Value** | **R Value** | **P Value** | **R Value** | **P Value** | **R Value** | **P Value** | **R Value** |
| Control | **0.04** | **0.31** | 0.13 | 0.23 | **<0.01** | **-0.46** | 0.32 | -0.15 |
| Spruce | 0.49 | 0.10 | **0.02** | **0.36** | **<0.01** | **-0.41** | **<0.01** | **-0.45** |
| Lodgepole | 0.91 | 0.02 | 0.09 | 0.25 | **<0.01** | **-0.43** | **<0.01** | **-0.43** |

Shading indicates significant correlations with a *P* value less than 0.05.
