# Supplementary material for: Effect of elevation, season and accelerated snowmelt on biogeochemical processes during isolated conifer needle litter decomposition
Source: PeerJ. 2021 Aug 10;9:e11926. doi: 10.7717/peerj.11926 (PMC8362670; doi:10.7717/peerj.11926)
Supplement: Supplemental Information 14 [file peerj-09-11926-s014.docx]

|  | **Year** | **Control** | **Spruce** | **Lodgepole** |
| --- | --- | --- | --- | --- |
| **Lower** | **2017** | R=0.2 P=0.2 | R=0.2 P= 0.6 | R=0.2 P=0.06 |
|  | **2018** | R=0.5 P=0.001 * | R=0.7 P=0.03 * | R=0.7 P=0.03 * |
|  | **2019** | R=0.2 P=0.1 | R=0.1 P=0.8 | R=0.3 P=0.7 |
| **Middle** | **2017** | R=0.2 P=0.5 | R=0.3 P=1 | R=0.2 P=0.4 |
|  | **2018** | R=0.6 P=0.001 * | R=0.4 P=0.06 | R=0.5 P=0.04 * |
|  | **2019** | R=0.1 P=0.7 | R=0.5 P=0.1 | R=0.4 P=0.2 |
| **Upper** | **2017** | R=0.3 P=0.6 | R=0.2 P=0.6 | R=0.2 P=1.0 |
|  | **2018** | R=0.2 P=0.2 | R=0.5 P=0.1 | R=0.2 P=0.4 |
|  | **2019** | R=0.3 P=0.8 | R=0.2 P=0.7 | R=0.2 P=0.4 |

*P* and R values represent ADONIS significance aggregated by date.
