# Supplementary material for: Effect of elevation, season and accelerated snowmelt on biogeochemical processes during isolated conifer needle litter decomposition
Source: PeerJ. 2021 Aug 10;9:e11926. doi: 10.7717/peerj.11926 (PMC8362670; doi:10.7717/peerj.11926)
Supplement: Supplemental Information 16 — Numeric values in each box represent the percent read abundance with all samples binned by elevation (Lower, Middle, Upper) and sampling date. Colors represent numerical ranges with higher numbers in red and lower numbers in blue. Differences between dates were determined using differential abundance comparisons of bacterial families in DESeq2. Significant differences (P <0.05) are highlighted in red with date comparisons indicated by the superscripts as follows: (1) May 2018 versus Jul 2018, (2) May 2018 versus Oct 2018, and (3) May 2018 versus May, Jun, or Jul 2019 depending on the elevation. [file peerj-09-11926-s016.pdf]

Lower

Phylum; Family

Phylum; Family	AUG 2017	OCT 2017	MAY 2018	JUL 2018	OCT 2018	MAY 2019	SEP 2019
[1,2] Bacteroidetes; Chitinophagaceae	28.3	35.4	44.1	15.7	13.5	23.1	31.2
Verrucomicrobia; DA101_soil_group	3	2.1	5.2	6.9	9.8	6.4	5.9
Proteobacteria; Oxalobacteraceae	14	6.4	4.3	5.5	2.5	4.1	5.7
[2] Proteobacteria; Comamonadaceae	2.8	3.3	3.5	2.7	2.5	2.9	3.2
Proteobacteria; Sphingomonadaceae	2.2	4.2	2.6	2.2	3.3	3.6	2.5
[1,2] Actinobacteria; Nocardioidaceae	1.9	3.3	1.5	3.1	3.7	2.9	2.4
Proteobacteria; Methylobacteriaceae	2.8	3.6	2.5	2.7	1.8	2.9	3.7
Proteobacteria; Xanthobacteraceae	2.5	0.8	1.9	2.6	2.9	3.4	2.5
Planctomycetes; Planctomycetaceae	2.2	1.1	2.1	3.2	3.1	2.5	1.3
Proteobacteria; Bradyrhizobiaceae	2.6	1.3	1.1	2.4	2.3	3	3
[1,2] Actinobacteria; Geodermatophilaceae	1.4	1.5	0.9	2.7	2.4	2.5	3.2
Planctomycetes; Tepidisphaeraceae	1.6	2.4	1.7	3.2	2.3	2.1	1.7
Acidobacteria; Blastocatellaceae_(Subgroup_4)	1.3	1.2	2	2.5	2.9	1.5	2.1
Actinobacteria; Micrococcaceae	1.7	1.8	1.2	2.3	2.8	1.7	1.6
Firmicutes; Planococcaceae	1.4	1.2	1.9	2	2.3	1.8	2

Middle

Phylum; Family	AUG 2017	OCT 2017	MAY 2018	JUL 2018	OCT 2018	JUN 2019	SEP 2019
[2] Bacteroidetes; Chitinophagaceae	14.9	17.9	17.2	9.5	4.7	13.4	17.3
Verrucomicrobia; DA101_soil_group	11.4	8.2	10.8	9.5	18.4	9.9	7.3
Proteobacteria; Bradyrhizobiaceae	4.3	3.1	3.4	3.9	4.2	4.7	4.4
Planctomycetes; Planctomycetaceae	3.9	3.1	4.2	3.7	5.7	4.2	2.6
Proteobacteria; Sphingomonadaceae	4.2	5.4	3.8	2.9	2.9	3.9	3.7
[2] Acidobacteria; Acidobacteriaceae_(Subgroup_1)	3.7	2.9	3.5	4.2	6.5	2.7	3.1
Acidobacteria; Solibacteraceae_(Subgroup_3)	3.6	3	3.2	4.1	4.1	3	3.6
Proteobacteria; Xanthobacteraceae	3.8	2.5	3.7	2.9	4.8	3.7	2.5
[2] Proteobacteria; Oxalobacteraceae	2.4	3.4	2.6	2.8	0.9	3.4	6.1
Planctomycetes; Tepidisphaeraceae	2.6	3	3	3	3.1	2.4	2.3
Proteobacteria; Acetobacteraceae	2.1	2.4	1.6	2.8	1.5	2.3	4.3
Firmicutes; Bacillaceae	1.7	1.9	1.6	2.2	1.9	2.1	2.4
[2] Proteobacteria; Comamonadaceae	1.8	2.2	2.1	1.9	1	2	2.3
[2] Proteobacteria; Methylobacteriaceae	1.7	2.2	1.8	1.9	0.8	2.1	2.9
Gemmatimonadetes; Gemmatimonadaceae	2.1	1	1.4	1.8	2.6	2.1	1.2

Upper

Phylum; Family	AUG 2017	OCT 2017	MAY 2018	JUL 2018	OCT 2018	JUL 2019	SEP 2019
[3] Acidobacteria; Acidobacteriaceae_(Subgroup_1)	9	8.9	11.4	12.9		8.7	7.8
[1] Bacteroidetes; Chitinophagaceae	9.2	7.8	11.3	5.7		9.8	12.5
[3] Verrucomicrobia; DA101_soil_group	8.5	9.3	12.2	10.2		8.1	6.4
Acidobacteria; Solibacteraceae_(Subgroup_3)	5.2	4.8	5.8	6.5		5.3	6
Planctomycetes; Planctomycetaceae	4.2	4.6	6.8	6.5		4.5	4.2
Proteobacteria; Bradyrhizobiaceae	5.1	3.7	4.3	4.3		6.4	6.6
[3] Proteobacteria; Acetobacteraceae	5.2	4.7	3.7	4.2		6.3	6.2
Proteobacteria; Xanthobacteraceae	3.4	3.1	3.5	3.3		3.6	4.1
Chloroflexi; HSB_OF53-F07	3.6	2.3	2.3	3.2		3.3	3
Chloroflexi; JG30a-KF-32	3.3	3	2.4	3.2		3.2	3
Planctomycetes; Tepidisphaeraceae	2.2	2.1	2.6	2.2		2.1	2.2
Proteobacteria; Sphingomonadaceae	2.4	3.8	2.1	1.8		1.7	1.7
Proteobacteria; Oxalobacteraceae	1.8	2.9	1.6	2		1.7	1.9
Firmicutes; ODP1230B8.23	2.2	1.9	1.4	1.6		2.3	1.9
Proteobacteria; Comamonadaceae	1.8	1.9	1.7	1.6		1.8	1.7
