# Supplementary material for: Effect of elevation, season and accelerated snowmelt on biogeochemical processes during isolated conifer needle litter decomposition
Source: PeerJ. 2021 Aug 10;9:e11926. doi: 10.7717/peerj.11926 (PMC8362670; doi:10.7717/peerj.11926)
Supplement: Supplemental Information 18 — Numeric values in each box represent the percent read abundance with all samples binned by elevation (Lower, Middle, Upper) and sampling date. Colors represent numerical ranges with higher numbers in red and lower numbers in blue. [file peerj-09-11926-s018.pdf]

Phylum

Lower

	AUG 2017	OCT 2017	MAY 2018	JUL 2018	OCT 2018	MAY 2019	SEP 2019
Ascomycota	36.9	44.9	26.9	23.5		50.1	67.4
Cercozoa	25.8	27	39.2	48.5		21.5	3.7
Basidiomycota	13.4	6.4	15.5	15		10	25.3
Incertae_Sedis	7	5.6	6.6	4		3.4	2
Phragmoplastophyta	5.7	2.8	1.6	0		9.9	0
Apicomplexa	0.2	7.1	0.3	0		0	0
Chytridiomycota	2.2	2.2	1.4	4.5		0.3	0.3
Annelida	0	0.4	4.5	0		4.3	0
Peronosporomycetes	4.1	0.2	0.4	1		0	0
Ciliophora	1.1	1	0.1	3.5		0.2	0.5
Ochrophyta	0.3	0.6	0.9	0		0.3	0.5
Arthropoda	0.7	0.2	0.9	0		0	0.3
Tardigrada	0.4	0.4	0.8	0		0	0
Chlorophyta_ph	0.7	0.5	0	0		0	0
Microsporidia	0.1	0.2	0.8	0		0	0

Middle

	AUG 2017	OCT 2017	MAY 2018	JUL 2018	OCT 2018	JUN 2019	SEP 2019
Ascomycota	43.8	47.4	31.8	59	16.5	56.1	52
Basidiomycota	10.2	15.8	32.3	24.2	6.5	8.7	42.2
Cercozoa	14.4	13.5	15.4	6.8	0	13.8	3.1
Phragmoplastophyta	15.2	5	11.7	0	74	8.4	0
Incertae_Sedis	3.8	4.8	3.1	4.1	2	3.8	0.4
Chlorophyta_ph	1.6	4.4	1.8	0	0	3.3	0
Arthropoda	4.5	2.5	0.8	0.7	0	0.2	0.1
Nematoda	2.1	1.6	0.1	1.2	1	0.1	0.3
Chytridiomycota	0.9	0.4	0.2	1.2	0	3.5	0.1
Ciliophora	1.4	0.6	0.1	0.5	0	0.6	0.5
Tardigrada	0.6	1.1	0.4	0.5	0	0.3	0.9
Klebsormidiophyceae	0.7	0	2.2	0.2	0	0.3	0
Ochrophyta	0.3	1.4	0.2	0.6	0	0.1	0.1
Schizoplasmodiida	0	0.3	0	1.1	0	0.2	0.1
Hyphochytriomycetes	0.1	0.2	0	0	0	0.4	0

Upper

	AUG 2017	OCT 2017	MAY 2018	JUL 2018	OCT 2018	JUL 2019	SEP 2019
Ascomycota	29.5	48.2	31.9			43.3	33
Phragmoplastophyta	18.3	3.5	6.7			19.8	22.4
Cercozoa	14.2	6.4	20.7			7.9	15.4
Basidiomycota	11.3	30.6	12.8			6.3	3.8
Chlorophyta_ph	5.6	0.8	11.1			5.4	0
Incertae_Sedis	5.1	2.4	6.8			9.5	5.9
Arthropoda	2.5	4.8	0.6			2.4	9.3
Cryptomycota	3.3	0.3	1.4			1.8	0
Ciliophora	2.6	0.3	2			0.4	0
Chytridiomycota	1.1	0.8	2.3			0.9	0
Nematoda	0.9	0.5	1.1			0	7.7
Annelida	1.8	0	0.2			0	0
Tardigrada	0.3	0	0.1			2.2	2
Schizoplasmodiida	1.1	0.3	0			0	0
Klebsormidiophyceae	0.2	0.5	1.4			0	0
